# Supplementary material for: Electric-field frictional effects in confined zwitterionic molecules
Source: Phys Chem Chem Phys. 2023 Jul 6;25(28):19037–45. doi: 10.1039/d3cp00914a (PMC10355170; doi:10.1039/d3cp00914a)
Supplement: CP-025-D3CP00914A-s004 [file CP-025-D3CP00914A-s004.pdf]

Electronic Supplementary Material (ESI) for Physical Chemistry Chemical Physics.
This journal is © the Owner Societies 2023

Electronic Supporting Information for Electric-field frictional effects in confined zwitterionic molecules

Melisa M. Gianetti,[*,†,⊥] Roberto Guerra,[‡] Andrea Vanossi,[¶,§] Michael Urbakh,[‖] and Nicola Manini[†]

†*Dipartimento di Fisica, Università degli Studi di Milano, Via Celoria 16, Milano 20133, Italy*

‡*Center for Complexity and Biosystems, Department of Physics, University of Milan, via Celoria 16, Milano 20133, Italy*

¶*CNR-IOM, Consiglio Nazionale delle Ricerche - Istituto Officina dei Materiali, c/o SISSA, Via Bonomea 265, 34136 Trieste, Italy*

§*International School for Advanced Studies (SISSA), Via Bonomea 265, 34136 Trieste, Italy*

‖*Department of Physical Chemistry, School of Chemistry, The Raymond and Beverly Sackler Faculty of Exact Sciences and The Sackler Center for Computational Molecular and Materials Science, Tel Aviv University, Tel Aviv 6997801, Israel*

⊥*Current affiliation: Institutt for maskinteknikk og produksjon, NTNU, Richard Birkelands vei 2B, 7034 Trondheim, Norway*

E-mail: melisamariel@gmail.com

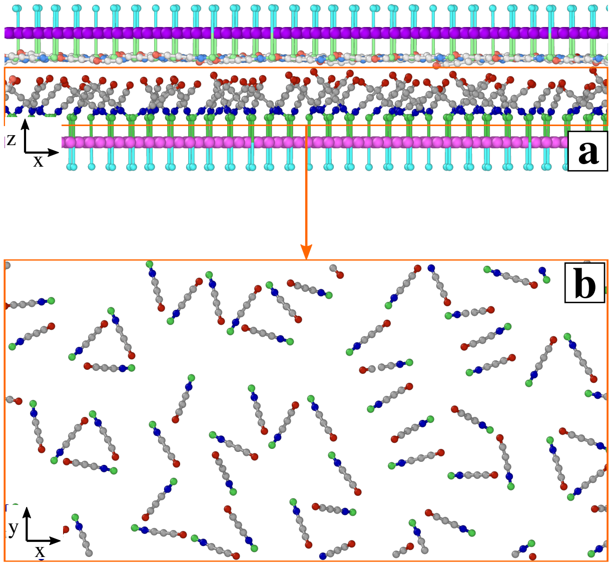

Figure S1: (a) Side view and (b) top view of a 4 nm y-thick slice of a snapshot of a simulation carried out with $E = 10\ \mathrm{V \cdot nm^{-1}}$, at $T = 300$ K. For better visibility, the top view includes the SUB chains only, i.e. the region inside the orange rectangle in panel (a).

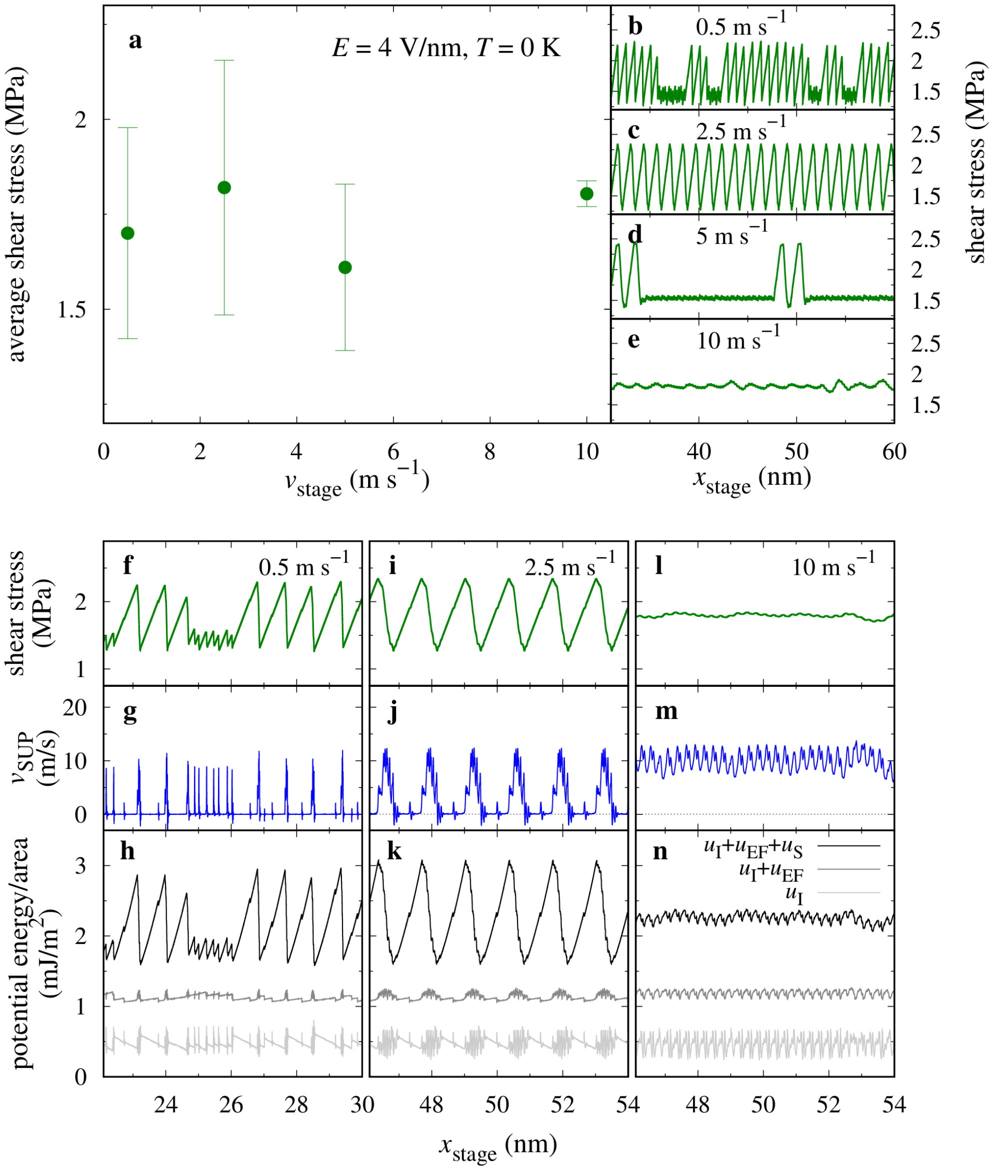

Figure S2: (a) Average shear stress as a function of v_{stage} for $E = 4\ \mathrm{V \cdot nm^{-1}}$, $T = 0$ K, and $L = 10$ MPa. (b-e) Shear-stress traces. Instantaneous (f,i,l) shear stress, (g,j,m) velocity of the SUP layer, and (h,k,n) per unit area potential-energy contributions as a function of x_{stage} for the corresponding velocities in panel (a). $u_{\mathrm{I}} = U_{\mathrm{I}}/A$ is the internal potential energy; $u_{\mathrm{EF}} = U_{\mathrm{EF}}/A$ is the potential energy for the interaction with the applied electric field; $u_{\mathrm{S}} = U_{\mathrm{S}}/A$ is the potential energy of the pulling spring.

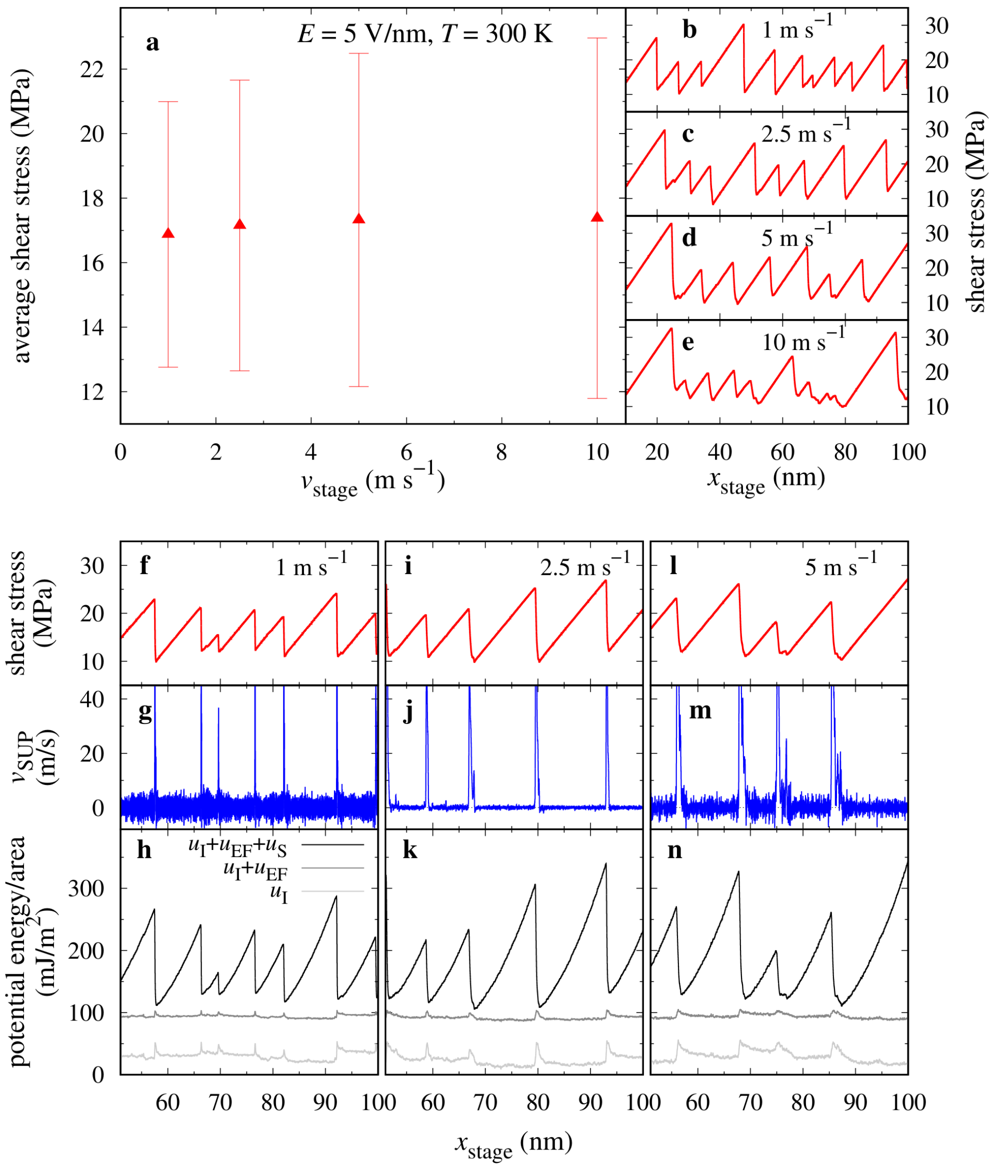

Figure S3: (a) Average shear stress as a function of v_{stage} for $E = 5\ \text{V}\cdot\text{nm}^{-1}$, $T = 300$ K and $L = 10$ MPa. (b-e) Shear-stress traces for the velocities reported in panel (a). (f,i,l) Shear stress, (g,j,m) instantaneous velocity of the SUP layer and (h,k,n) per unit area potential energy contributions as a function of x_{stage} for the corresponding velocities in panel (a). In the analysis of the potential energy contributions (panels h,k,n), thermal noise affects the data heavily: to discern readable energy signals we apply a Gaussian smoothing with width of 0.05 nm. To mitigate the thermal noise in the SUP velocity we evaluate its average value by means of finite differences of the SUP position over the time corresponding to the driving stage advancing by 0.05 nm.

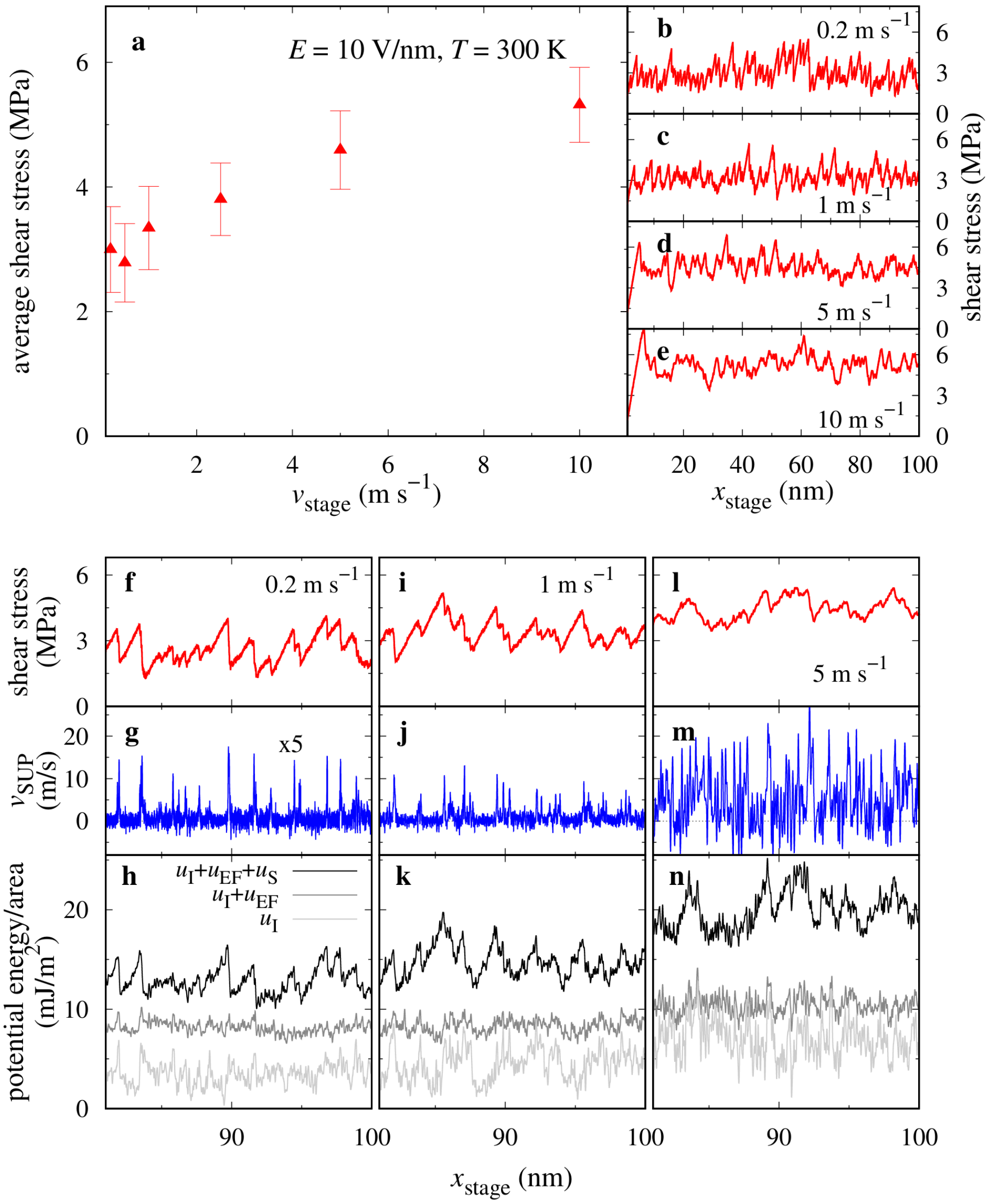

Figure S4: (a) Average shear stress as a function of v_{stage} for $E = 10\ \text{V} \cdot \text{nm}^{-1}$, $T = 300$ K and $L = 10$ MPa. (b-e) Shear traces for a few of the velocities reported in panel (a). (f,i,l) Shear stress, (g,j,m) instantaneous velocity of the SUP layer and (h,k,n) per unit area potential energy contributions as a function of x_{stage} for the corresponding velocities in panel (a). Thermal noise has been mitigated like in Figure S3. The intense electric field keeps the SUP layer of chains flat to the point of remaining nearly "frozen", thus preventing the entanglement of SUB chains. Friction traces at the lowest velocities do show some hints of stick points (Figure S4f, i) and eventually at low speed friction seems to depend only weakly on the sliding velocity, Figure S4a. Stick-slip features are visible in the SUP velocity and total potential energy, while they are harder to detect in the internal contribution U_{I}. Even at $T = 300$ K, signs of the unexpected slight decrease of U_{I} during stick can be detected near $x_{\text{stage}} \simeq 82$ nm and $x_{\text{stage}} \simeq 90$ nm, similar to that discussed for $T = 0$ in the main text.

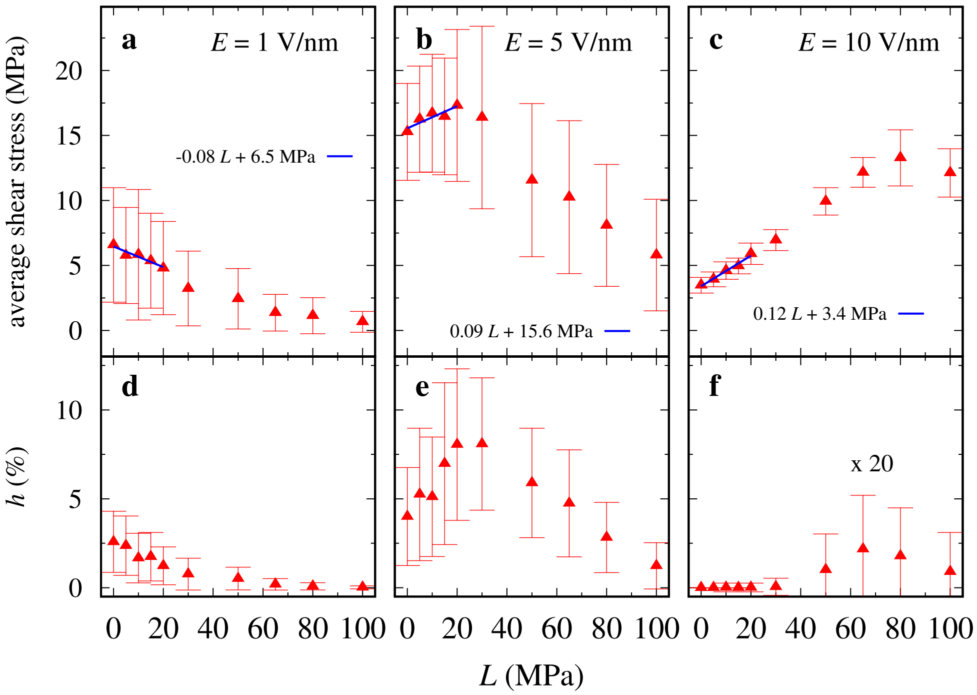

Figure S5: (a-c) Average shear stress (same as Fig. 7 of the article) and (d-e) average hooking fraction h (our strategy for quantifying the degree of interlocking defined in the Supporting Information of Ref. 1) as a function of the applied load L at $T = 300$ K. Blue lines are linear fits of the small-load (up to 20 MPa) data. The resulting differential friction coefficients are: $\mu_d = -0.08$ for $E = 1\ \mathrm{V \cdot nm^{-1}}$; $\mu_d = 0.09$ for $E = 5\ \mathrm{V \cdot nm^{-1}}$; $\mu_d = 0.12$ for $E = 10\ \mathrm{V \cdot nm^{-1}}$.

SI Movies

Each of the SI movies reports 3 ns (corresponding to a 15 nm advancement of the stage) of a MD simulation. In simulation time, the frame rate is 1 frame every 20 ps. In running time, the frame rate is 10 frames per second. For clarity, like in Fig. S1, the movies only include a 5 nm y-thick slice of the simulation cell (whose entire y-side is 14.41 nm). One of the particles of the SUP layer is drawn of bigger size and lighter color to improve the visibility of the advancement of this layer.

- **Movie1.mp4**: 3 ns of a $E = 2\ \mathrm{V \cdot nm^{-1}}$, $v_{\mathrm{stage}} = 5\ \mathrm{m \cdot s^{-1}}$, $T = 300$ K simulation, also reported in the snapshots of Figure 3 of the main text;

- **Movie2.mp4**: 3 ns of a $E = 5\ \mathrm{V \cdot nm^{-1}}$, $v_{\mathrm{stage}} = 5\ \mathrm{m \cdot s^{-1}}$, $T = 300$ K simulation, also reported in the snapshots of Figure 4b,c,e,f of the main text;

- **Movie3.mp4**: 3 ns of a $E = 10\ \mathrm{V \cdot nm^{-1}}$, $v_{\mathrm{stage}} = 5\ \mathrm{m \cdot s^{-1}}$, $T = 300$ K simulation, also reported in the snapshots shown in Figure S1 and in the friction trace shown in Figure S4d,l of the present SI.

References

(1) Gianetti, M. M.; Guerra, R.; Vanossi, A.; Urbakh, M.; Manini, N. Thermal Friction Enhancement in Zwitterionic Monolayers. *J. Phys. Chem. C* **2022**, *126*, 2797–2805.
